# Food craving in daily life: comparison of overweight and normal‐weight participants with ecological momentary assessment

**DOI:** 10.1111/jhn.12693

**Published:** 2019-08-20

**Authors:** A. Roefs, B. Boh, G. Spanakis, C. Nederkoorn, L. H. J. M. Lemmens, A. Jansen

**Affiliations:** ^1^ Faculty of Psychology & Neuroscience Maastricht University Maastricht The Netherlands; ^2^ Department of Data Science and Knowledge Engineering Maastricht University Maastricht The Netherlands

**Keywords:** ecological momentary assessment, experience sampling, food craving, obesity, overweight

## Abstract

**Background:**

The present study examined food cravings in daily life by comparing overweight and normal‐weight participants right before eating events and at non‐eating moments. It was hypothesised that overweight participants would have (i) more frequent, (ii) stronger and (iii) a greater variety of high‐caloric palatable food cravings, and also would (iv) consume more high‐caloric palatable foods, than normal‐weight participants.

**Methods:**

Ecological momentary assessment (EMA) was used to assess food craving strength and frequency, variety of specific food cravings, and food intake. Fifty‐seven overweight and 43 normal‐weight adult participants were assessed at eating events and at an average of eight random non‐eating moments per day for 2 weeks. Foods were categorised as: high‐caloric high palatable foods (HCHP), fruits and salads, staple food dishes and sandwiches, and soups and yoghurts.

**Results:**

Overweight participants reported more frequent HCHP food cravings specifically at non‐eating moments than did normal‐weight participants. Normal‐weight participants reported more food cravings for staple foods, specifically at eating events. Moreover, overweight participants craved a greater variety of HCHP foods than normal‐weight participants at both eating events and random non‐eating moments. No other significant between‐group differences were found.

**Conclusions:**

The results highlight the importance for obesity interventions (i) to specifically target high‐caloric palatable food cravings that are experienced during the day and are not tied to eating moments and (ii) to aim for a reduction in the variety of high‐caloric palatable food cravings. It might be fruitful to deliver treatment aimed at reducing cravings via mobile devices because this allows for easy individual tailoring and timing of interventions.

## Introduction

People eat not only to satisfy homeostatic hunger, but also to satisfy cravings and hedonic hunger 1, 2. Food cravings are common 3, and people often crave foods that are high in calories and low in nutritional value 4, 5. High‐caloric food intake has been associated with weight gain and obesity 6. Furthermore, sensitivity to the rewarding properties 7 and the reinforcing value 8 of palatable foods is stronger for people with a higher body mass index (BMI) than for those with a lower BMI. The present study aimed to investigate food cravings and intake of overweight (BMI ≥ 25 kg/m^2^) and normal‐weight (18.5 kg/m^2^ ≤ BMI < 25 kg/m^2^) people in daily life by means of ecological momentary assessment (EMA).

Previous research on food craving, mainly relying on retrospective self‐report (questionnaire) assessment, has found that overweight people have more frequent specific food cravings, mainly for high‐caloric foods, compared to normal‐weight people 4, 9, 10, 11. In addition, a meta‐analysis has shown that craving and food‐cue reactivity are significant predictors of eating behaviour and body weight 12, as well as of a reduction of perceived self‐regulatory success in dieting 13. Accordingly, an increased food craving appears to be an important aspect of obesity. However, retrospective self‐report assessment is subject to memory recall biases: more recent and more emotionally salient memories are disproportionately often recalled 14, 15. Investigating food cravings as they occur in daily life using EMA could lead to more ecologically valid insights.

During EMA, participants receive prompts on a mobile device (e.g. smartphone) several times a day to answer questions, for example regarding mood, social circumstances and food cravings, and/or are instructed to answer questions on their phone in predefined situations (e.g. when about to eat something). EMA has the advantage that data are obtained in the moment and in daily life (ecological validity) and are not affected by retrospective memory or response bias 14. Another advantage is that participants provide multiple assessments of the included variables, allowing researchers to analyse how the variables develop over time within a participant. Thus, ‘EMA aims to minimize recall bias, maximize ecological validity and allow study of microprocesses that influence behavior in real‐world contexts’ 15. EMA conducted via an electronic device may be especially suitable to assess food cravings because of the relatively short duration of such cravings 16.

EMA studies on food craving have increasingly been published in recent years. It was found that food cravings for sweet and salty snacks increased over the day, with a reduced coherence with hunger 17. In addition, a study focusing on snacks found that most reported snacks were high‐caloric (86%) and that craving intensity was positively associated with snack consumption 18. Studies focusing on dieters found that 17% 5 to 50% 19 of daily life food cravings resulted in dietary lapses. Interestingly, the strength and the frequency of food cravings were not related to dietary restraint 5, although dieters were more likely to give in to food temptations if the craving to eat was stronger 19. With regard to body weight, it was unexpectedly found that a group of people with obesity reported fewer unresisted food cravings compared to a lean group. However, within the obese group, the fraction of unresisted food craving was positively associated with BMI 20. Because that study employed only event‐related sampling (i.e. self‐initiated measurement at eating occasions), this may be a result of BMI‐related under‐reporting.

Another aspect of food craving relates to the variety of craved foods. Although previous research has linked food *intake* variety to obesity 21, 22, 23, not much is known about food craving variety. Sensory‐specific satiety has been suggested to explain the link between intake variety and obesity 24, 25: satiety occurs separately for each of the sensory characteristics of different kinds of foods. Accordingly, when a large variety of foods is consumed, it will take longer for satiety to set in, which may lead to increased intake 26. In general, food cravings and the subsequent intake of these craved foods are highly positively associated 4, 11, 27, 28, with one possibility being that food craving variety is also related to obesity.

Taken together, the present study investigates how food craving frequency, strength and variety, as well as food intake, are related to weight status. The study addresses the following hypotheses: overweight participants (i) report more frequent and (ii) stronger food cravings for high‐caloric palatable foods, (iii) they crave a greater variety of high‐caloric palatable foods and (iv) they consume more high‐caloric palatable foods compared to normal‐weight participants. In addition, the association between specific food cravings and food intake is investigated. Food craving frequency, strength and variety are investigated and compared separately for eating events (i.e. that were about to occur) and non‐eating moments. There are no specific *a priori* hypotheses about differences between eating events and non‐eating moments in terms of food craving frequency, strength or variety.

## Materials and methods

The data reported in the present paper were collected as part of a large EMA paper investigating predictors of eating behaviour in daily life. A previous paper 29 focused on cognitions and emotions. One other previous paper focused on time‐lagged network analysis of associations between food craving, emotions and eating events 30. The study was approved by the Ethical Committee of the Faculty of Psychology and Neuroscience of Maastricht University.

### Participants

Participants were recruited via flyers distributed in the university, academic hospital, health centres, supermarkets and household fairs. Advertisements were placed in newspapers, on Facebook and on several other websites. Inclusion criteria for participation in the study were: (i) BMI between 18.5 and 40; (ii) in possession of an iPhone; (iii) not on a supervised diet; (iv) no medical conditions that could affect eating behaviour; and (v) not pregnant. In total, 67 overweight and 44 normal‐weight participants applied. Note that, in the present paper, the term ‘overweight’ also refers to obese (BMI > 30 kg/m^2^) participants. Six overweight participants were lost to dropout and five participants (*n *=* *4 overweight; *n *=* *1 normal‐weight) were excluded from participation because of <10% compliance with the EMA protocol. Two participants provided a self‐reported BMI measurement at initial screening that was below 40, although they turned out to have a BMI of 40.8 and 45.7 upon actual BMI measurement. These participants were included in the final sample to increase the overall study power.

The final sample consisted of 57 overweight (seven male) and 43 normal‐weight (five male) participants. There were no significant differences in BMI between the post‐EMA measurement [mean (SD) overweight: 30.2 (4.2); normal‐weight: 22.2 (1.5)] and pre‐EMA measurement [mean (SD) overweight: 30.3 (4.3); normal‐weight: 22.1 (1.5)] for the overweight group (*t*_56_ = 1.07, *P *=* *0.29), nor for the normal‐weight group (*t*_42_ = 1.22, *P *=* *0.23). Furthermore, education level (χ^2^ = 2.3, *P *=* *0.31) and sex ratio (χ^2^ < 0.01, *P *>* *0.99) did not differ significantly between groups. The mean (SD) age of participants was 31.2 (10.0) years for the overweight group and 32.1 (10.6) years for the normal‐weight group, with no significant difference between overweight and normal‐weight participants (*t*_98_ = 0.43, *P *=* *0.67).

### General ecological momentary assessment protocol

Participants used an iPhone application to complete assessments of variables related to eating behaviour (food cravings, food intake, emotions, cognitions preceding food intake, physical locations and activities). The present study focuses on the measures of craving and intake only. Food craving was assessed both immediately prior to each eating event, and after receiving an automated notification on the participant's iPhone requesting an assessment (non‐eating moment assessments; signal‐contingent sampling). For eating event sampling, the participant was instructed to initiate an EMA questionnaire on his/her iPhone just prior to each eating event. Non‐eating moment assessments were randomly distributed over the waking day, occurring once per 2‐h time window, resulting in approximately eight prompts per day. Sleeping and waking times were modifiable in the app.

### Measures of food craving and intake

#### At eating events and non‐eating moments

At each assessment, strength of food craving was indicated on a visual analogue scale ranging from 0 to 100 by answering the question: ‘How strong is your craving to eat?’. Note that, in the Dutch language, there is no clear distinction between ‘food craving’ and ‘food desire’. Importantly, on each occasion, participants were asked whether they craved one or more *specific* foods (yes or no). If the participant answered ‘yes’, this was counted as an occurrence of specific craving. Moreover, if the participant answered ‘yes’, an overview of 19 food‐icons was presented, reflecting different types of foods (Table 1). These 19 food types were chosen to reflect the most common food types eaten in a typical Dutch diet. The participant was instructed to select icons of food that most closely resembled the craved food, and could select multiple foods. For example, if a participant craved salty sticks, the instruction was to select the icon ‘chips’ (i.e., representing the category ‘salty snacks’). Participants received printed manuals of the app functionality. If the participant answered ‘no’ to the question whether specific craving occurred, this was *not* counted as a case of specific craving, and only the craving strength‐score was analysed.

**Table 1:** Food icons allocated to each food category

Food category	Represented by these icon(s)
High‐caloric high palatable (HCHP)	Hamburger, muffin, cookies, candy bar, candy, dishes with a side of fries, chips, pizza, cake, ice cream
Fruits and salads	Salad, apple
Staple dishes and sandwiches	Sandwiches, pasta, cornflakes, dishes with a side of potatoes, dishes with a side of rice
Soups and yoghurts	Yoghurt, soup

#### At eating events only

At eating events, an additional question, ‘What are you about to eat?’, was asked, and participants were asked what they were about to eat with the same overview of 19 food‐icons used for the food craving question. Multiple food‐type selections were possible. To verify that food intake took place, participants were asked to take a picture of the food. Note that food quantity was not measured.

### Procedure

Prior to starting, the participant followed 1 day of training to become familiar with the app and EMA procedure, as well as to resolve technical issues. In addition, the participant was instructed to obtain measurements of body weight and height. These measurements were conducted by a healthcare professional or a researcher for 89% of participants (11% self‐reported height and weight). After obtaining these measurements, the participant was enrolled in a 2‐week EMA period, during which the participant could ask the experimenter for help when experiencing technical or other difficulties. At day 3 or 4, the participant was contacted by phone to check for problems and, at day 8, an e‐mail was sent to motivate the participant to keep up the good work. After the EMA period, the participant was asked to have body weight measured again under identical circumstances as for the first measurement. After obtaining the measurement, the participant was debriefed about the purposes of the study and received a €50 voucher.

### Statistical analysis

The 19 food types were first sorted into four main categories: (i) high‐caloric, high palatable foods (HCHP); (ii) fruits and salads; (iii) sandwiches and foods often served as staple parts of a dinner (staple); and (iv) soups and yoghurts (Table 1).

For each participant, the total number of specific food cravings per food category was summed separately for eating events and non‐eating moments. Percentages were then computed by dividing the number of specific food cravings per food category per type of measurement (eating event or non‐eating moment) by the total number of specific food cravings (eating events plus non‐eating moments). Note that one normal‐weight participant who did not report any specific food cravings was not included in these analyses because this would lead to a division by zero. It was also determined, for each participant, how often a food from each of the four categories was eaten by computing percentages relative to the total number of food products that were eaten by each participant.

Craving strength was only scored once during each EMA assessment. In the case of multiple food cravings, this craving strength score was considered to apply equally to all selections for that particular assessment. For each participant, 10 craving strength scores were computed, by averaging craving strength scores of the four food categories and for nonspecific cravings, separately for eating events and non‐eating moments.

In addition, variety of food craving and food intake were determined. To increase comparability between the number of foods in the HCHP category (which concerns 10 food‐types) and in the other three categories (which concern two, five and two food‐types, respectively), data of these latter three categories were summed (referred to as ‘other foods’). For each participant, and separately for each food category (HCHP or ‘other foods’), the number of *different* food cravings and the number of different consumed foods during the 2‐week EMA period were determined. These numbers were then divided by the total number of foods available in each category and multiplied by 100. This provided a percentage, where 100% indicates all foods of a category were craved for or consumed. Note that the one normal‐weight participant who did not report any specific food cravings was not included in the analyses on food craving variety. Finally, the match between food cravings and food intake was analysed. At eating event assessments, specific food cravings and eating were considered a match if at least one of the craved foods was also consumed at that eating event. Two normal‐weight participants and one overweight participant who did not report any specific food cravings at eating moments were not included in these analyses. The alpha‐level was set to the standard α = 0.05.

Note that our sample size was sufficiently large (*n *=* *100) to assume a normal distribution according to the central limit theorem, which requires a minimum of 20 degrees of freedom for error 31, provided that there are no outliers. Because some of our variables did contain some outlier values (>3 × interquartile range), we also conducted nonparametric tests (independent‐samples Mann–Whitney *U* tests) for all comparisons. All group differences that were significant using parametric tests remained significant when analysed using nonparametric tests. One trend‐significant (i.e. 0.05 > *P *< 0.10) finding of a parametric test (*P *=* *0.06) (Table 2) just reached significance with a nonparametric test (*P *=* *0.049). Accordingly, the pattern of results was independent of the use of parametric versus nonparametric tests. Finally, Cohen's *d* for the sample (*d*_s_) is reported as a measure of effect size 32.

**Table 2:** Craving strength scores and comparisons between overweight and normal‐weight participants for each food category and for nonspecific cravings

	Food categories	Overweight participants		Normal‐weight participants		Between‐group comparisons				
		Mean	(SD)	Mean	(SD)	*t*	d.f.	*P*	*d*_s_	95% CI diff
Eating events	Nonspecific	53.74	(16.16)	54.92	(16.93)	0.35	97	0.72	0.07	−5.46 to 7.82
	HCHP	59.39	(17.68)	60.64	(16.19)	0.35	91	0.73	0.07	−5.85 to 8.34
	Fruits and vegetables	58.37	(18.65)	57.78	(17.41)	0.13	64	0.89	0.03	−9.50 to 8.31
	Staple foods	69.81	(13.94)	71.28	(12.30)	0.49	76	0.63	0.11	−4.53 to 7.46
	Soups and yoghurts	61.07	(20.67)	64.09	(17.66)	0.56	49	0.58	0.17	−7.87 to 13.90
Non‐eating moments	Nonspecific	23.86	(11.33)	28.26	(11.10)	1.94	98	0.06	0.39	−0.10 to 8.90
	HCHP	53.77	(19.76)	57.65	(19.17)	0.93	87	0.36	0.20	−4.46 to 12.23
	Fruits and vegetables	57.08	(22.29)	54.10	(23.23)	0.44	43	0.66	0.13	−16.66 to 10.71
	Staple foods	64.13	(19.70)	67.43	(18.86)	0.73	73	0.47	0.17	−5.67 to 12.26
	Soups and yoghurts	57.02	(26.87)	62.26	(21.22)	0.69	39	0.50	0.21	−10.23 to 20.71

Craving strength was scored on a visual analogue scale from 0 (no craving at all) to 100 (very strong craving). Regarding the 95% confidence intervals: a negative difference score means that overweight participants scored higher than did normal‐weight participants, whereas a positive difference score means that overweight participants scored lower than did normal‐weight participants. Variation in d.f. is the result of a varying number of participants reporting cravings for the different food categories.

95% CI diff, 95% confidence interval of the between‐group difference; *d*_s_, Cohen's *d* for the sample; HCHP, high‐caloric highly palatable.

## Results

### Compliance

Participants completed a mean (SD) of 81.44% (10.19%) of all non‐eating moment assessments during the 2‐week EMA‐period and registered a mean (SD) of 55.28 (17.86) eating events. Note that, at each eating event, multiple food types could be selected.

### Overall frequency of cravings for specific foods

#### Eating events

Cravings for specific foods were reported for a mean (SD) of 40.72% (*SD *=* *30.46%) of all eating events for overweight participants and 38.89% (28.85%) of all eating events for normal‐weight participants, with no significant difference between groups [*t*_98_ = 0.30, *P *=* *0.76, *d*_s_ **= 0.06, 95% confidence interval (CI) = −13.76 to 10.11].

#### Non‐eating moments

On average, a mean (SD) of 14.35% (11.94%) of non‐eating moments of overweight participants and 14.30% (13.38%) of non‐eating moments of normal‐weight participants contained a craving for specific foods, with no significant difference between groups (*t*_98_ = 0.02, *P *=* *0.99, *d*_s_ *=* 0.004, 95% CI = −5.09 to 4.99).

### Frequency of cravings for specific food categories

**Figure 1:**
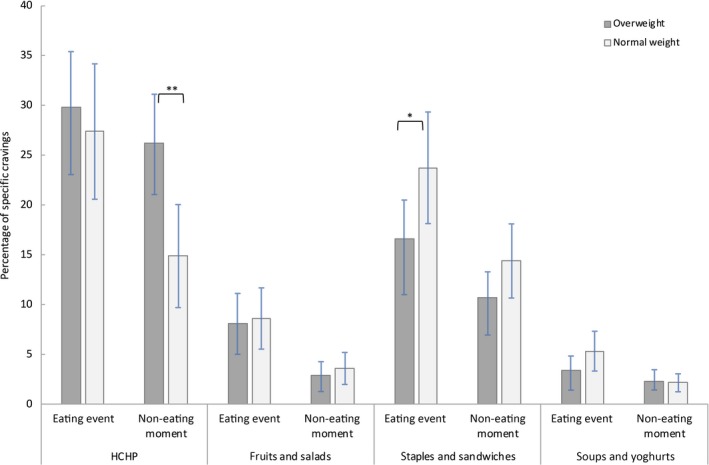
Average percentage of specific cravings for each food category for overweight and normal‐weight participants. Data are presented per food category [high‐caloric highly palatable (HCHP) foods, fruits and salads, staple dishes and sandwiches, and soups and yoghurts]. For each participant, percentages were computed by dividing the number of specific food cravings per food category per type of measurement (eating event or non‐eating moment) by the total number of reported specific food cravings (eating events plus non‐eating moments). Error bars represent the 95% confidence interval of the mean (±1.96 × SEM). **P ≤ 0.01, *P ≤ 0.05.

Food categories were: (i) HCHP foods; (ii) fruits and salads; (iii) staple foods; and (iv) soups and yoghurts. Overweight participants reported significantly more specific HCHP food cravings at non‐eating moments than normal‐weight participants (*t*_97_ = 3.08, *P *=* *0.003, *d*_s_ *=* 0.63, 95% CI = −18.70 to −4.03). In addition, normal‐weight participants reported significantly more staple food cravings at eating events than overweight participants (*t*_97_ = 2.13, *P *=* *0.04, *d*_s_ **= 0.43, 95% CI = 0.48 to 13.86). None of the other comparisons reached significance (all *t*_97_ < 1.64, all *P *>* *0.10) (Figure 1).

### Food craving strength scores

Craving strength scores were analysed separately for each of the four food categories and for nonspecific food cravings. None of the between‐group comparisons reached significance. Relevant statistics are provided in Table 2.

### Foods consumed at eating events

The frequency of reported food intake for each of the four food categories was analysed. None of the between‐group comparisons reached significance. Relevant statistics are provided in Table 3.

**Table 3:** Intake per food category expressed as a percentage of the total number of reported consumed foods, presented for overweight and normal‐weight participants

Food categories	% Intake per food category				Between‐group comparisons			
	Overweight		Normal‐weight					
	Mean	(SD)	Mean	(SD)	*t*_98_	*P*	*d*_s_	95% CI diff
HCHP	25.50	(13.22)	23.44	(11.39)	0.82	0.42	0.16	−7.06 to 2.94
Fruits and salads	16.18	(10.71)	16.07	(11.56)	0.05	0.96	0.01	−4.55 to 4.34
Staple dishes and sandwiches	49.70	(12.40)	50.36	(13.45)	0.26	0.80	0.05	−4.49 to 5.82
Soups and yoghurts	8.62	(5.95)	10.12	(8.15)	1.07	0.29	0.22	−1.30 to 4.30

Regarding the 95% confidence intervals, a negative difference score means that overweight participants scored higher than normal‐weight participants, whereas a positive difference score means that overweight participants scored lower than normal‐weight participants.

95% CI diff, 95% confidence interval of the between‐group difference; *d*_s_, Cohen's *d* for the sample; HCHP, high‐caloric highly palatable.

### Variety in food cravings and food intake

**Figure 2:**
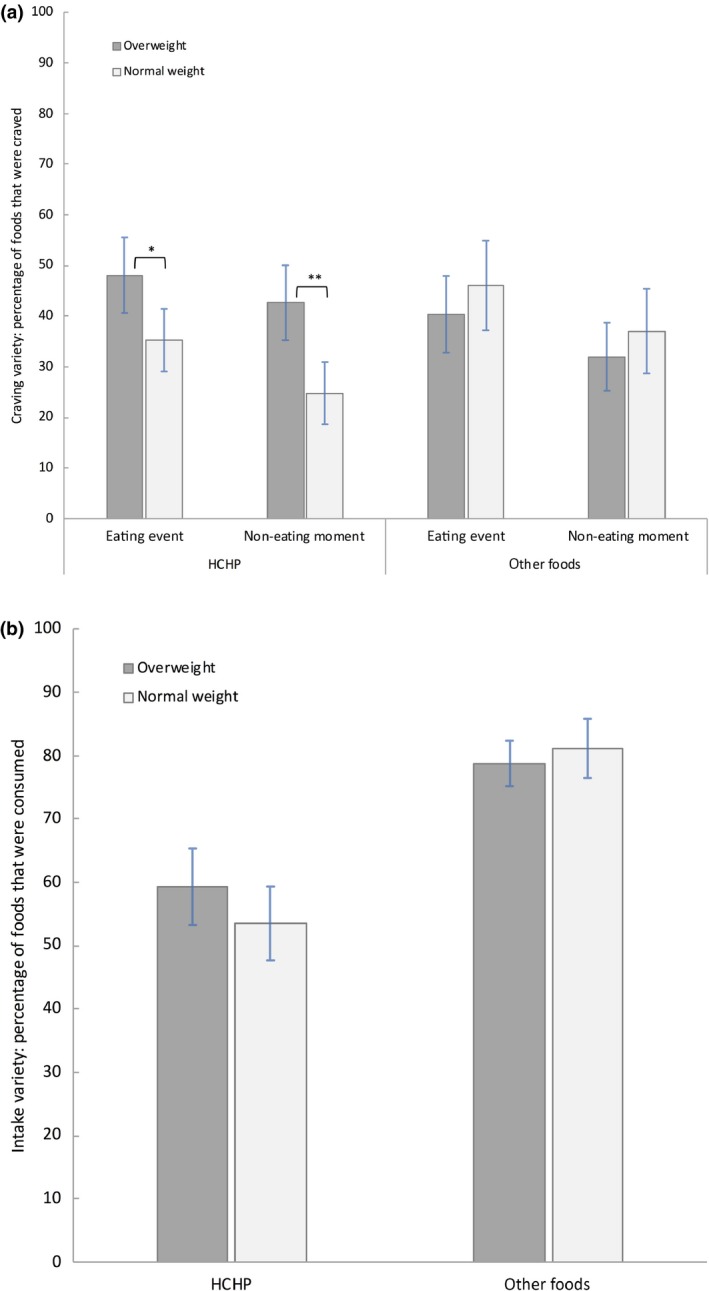
(a) Craving variety. (b) Intake variety. Variety of food cravings (a) and variety of food intake (b) were computed for each participant by dividing the number of different craved (a) and consumed (b) foods by the total number of foods available within each category (multiplied by 100 to arrive at a percentage). The total number of available foods was identical for food cravings and food intake. For food craving variety, computations were done separately for eating events and non‐eating moments. The ‘other foods’ category is a combination of the three non‐high‐caloric highly palatable (HCHP) food categories. The HCHP food category consists of 10 different food‐types, whereas the ‘Other’ foods category consists of nine different food‐types. Error bars represent the 95% confidence interval of the mean (±1.96 × SEM). **P ≤ 0.01, *P ≤ 0.05.

Overweight participants reported significantly more variety in HCHP food cravings at eating events (*t*_96.87_ = 2.59, *P *=* *0.01, *d*_s_ *=* 0.50, 95% CI = −22.65 to −3.02) and at non‐eating moments (*t*_96.90_ = 3.65, *P *<* *0.001, *d*_s_ *= *0.70, 95% CI = −27.60 to −8.14) than normal‐weight participants. For the ‘other foods’ category, neither the comparison for eating events (*t*_97_ = 0.96, *P *=* *0.34, *d*_s_ *=* 0.19, 95% CI = −6.11 to 17.47), nor the comparison for non‐eating events reached significance (*t*_97_ = 0.94, *P *=* *0.35, *d*_s_ *=* 0.19, 95% CI = −5.68 to 15.82) (Figure 2a).

Variety of food intake did not differ significantly between normal‐weight and overweight participants, for both HCHP foods (*t*_98_ = 1.32, *P *=* *0.19, *d*_s_ *=* 0.27, 95% CI = −14.53 to 2.91) and ‘other foods’ (*t*_98_ = 0.81, *P *=* *0.42, *d*_*s*_ *=* 0.16, 95% CI = −3.48 to 8.24) (Figure 2b).

### Matches between specific food cravings and eating

For the percentage craving‐intake matches, there were no significant differences [mean (SD)] between overweight [90.83% (15.80%)] and normal‐weight [92.39% (13.78%)] participants (*t*_95_ = 0.51, *P *=* *0.61, *d*_s_ *= *0.10, 95% CI = −4.55 to 7.68).

## Discussion

In the present study, several aspects of food cravings were compared between overweight and normal‐weight participants. The main findings related to our four specified hypotheses include: (i) Overweight participants reported more frequent specific high‐caloric (HCHP) food cravings specifically at non‐eating moments than did normal‐weight participants, whereas normal‐weight participants reported more frequent specific cravings for meal‐type foods and sandwiches (staple foods) at eating events than did overweight participants. (ii) No significant differences were observed between overweight and normal‐weight participants for food‐craving strength scores. (iii) Overweight participants reported a wider variety of specific HCHP food cravings than normal‐weight participants, both at eating events and at non‐eating moments. No significant between‐group differences were observed for variety in specific food cravings for other foods. In addition, no significant between‐group differences were observed for variety in food intake. (iv) No significant differences between overweight and normal‐weight participants were observed for self‐reported actual food intake for any food type. Finally, a strong association was observed between the type of food that was craved and that was actually eaten at eating events. This association was not significantly different between overweight and normal‐weight participants.

Overweight participants reported more frequent cravings specifically for HCHP foods, specifically at non‐eating moments. In the Western obesogenic environment 33, food is widely available, and so a wide variety of cues can be conditioned to trigger cravings for food, such as recurring contexts and activities 34, 35, 36, 37. Possibly, overweight participants associated more aspects of the environment with the intake of HCHP foods than normal‐weight participants, leading to more frequent high‐caloric food cravings. Notably, these findings fit well with recent research showing that eating at an unintended time significantly predicted worse weight loss outcomes in overweight people 38. Food cravings of normal‐weight participants were more often tied to eating events, including breakfast, lunch and dinner, and food craving occurred mostly for foods that are typically consumed at those types of eating events. These findings expand previous research showing that overweight people have an increased craving for (high‐caloric) food 4, 9, 10, 11. Such increased craving appears to occur mostly for HCHP foods, and to occur at random times during the day and is not tied to eating moments. Note that these findings were not paralleled in analyses of the *strength* of food cravings. Note also that the strength scores of food cravings were only medium (mostly between 50 and 60) (Table 2) and might not compare to how craving has been defined in previous studies (i.e. as being clearly distinct from desires). As noted before, in the Dutch language, there is no clear distinction between food‐desire and food‐craving. This might explain the relatively frequent occurrence of food cravings compared to some previous studies.

The association of food craving and subsequent eating in the present study was strong, and similar for overweight and normal‐weight participants: about nine in 10 times, on average, craving for specific foods at eating moments was followed by the intake of *at least one* type of food that was craved. This finding is in line with findings of a previous laboratory study of overweight participants, in which food cravings for certain types of high‐caloric foods resulted in significantly more intake of that type of food than of other types of foods 27. It is also in line with findings of a questionnaire study reporting that craving for foods with certain sensory aspects (e.g. sweetness) was followed by intake of foods with those specific sensory aspects, irrespective of participants’ BMI 4. This strong connection is likely partly a result of the timing of the measurement, in that participants were about to eat and the food was in front of them.

In line with the hypothesis, overweight participants reported more varied HCHP food cravings than normal‐weight participants, at eating events and at non‐eating moments. Thus, it appears that the more frequent occurrence of specific HCHP food cravings for overweight participants does not reflect craving for particular HCHP foods (e.g. chocolate lovers), but it likely reflects craving for many different HCHP foods. Note that we did not obtain hunger ratings, and therefore cannot exclude the possibility that differences in craving variety are driven by differences in hunger. For food intake variety, however, contrary to previous research 21, 22, 23, no significant between‐group difference was observed. It should be noted that, in the present study, food intake was often not accompanied by a specific food craving. That is, specific food cravings occurred for only about 40% of the eating events, which may explain the discrepancy in results for craving versus intake variety.

In the present study, there were no overall differences in the frequency of eating events between overweight and normal‐weight participants. This could indicate a selective under‐reporting of eating events in the overweight group. Previous research found that under‐reporting of food intake was associated with BMI 39, 40. As with questionnaire‐based research in general, social desirability could have influenced the reporting of eating events 41. Alternatively, overweight participants may have been more conscious about their food intake during the 2‐week EMA period, leading to more intake control efforts. Finally, food quantity was not assessed in the present study. Although the number of reported eating events in the present study is comparable to other EMA research on food intake 42, it is possible that overweight and normal‐weight people differ in the amount of food consumed per eating event.

Taken together, EMA of eating events and non‐eating moments provided us with insights into the specificity, strength and variety of food cravings and how these relate to food intake and as they occur in daily live. This would not have been possible with the use of retrospective measures. Compared to normal‐weight participants, overweight participants’ high‐caloric food cravings more commonly occurred throughout the day, and were not tied to self‐reported eating events. Normal‐weight participants more often reported staple food cravings when about to eat compared to overweight participants. Moreover, both at eating events and otherwise throughout the day, food cravings were more varied for overweight than for normal‐weight participants. It should be kept in mind that our sample was predominantly female, and the results may be different for males. Also, for future research, it is of interest to include a group of exclusively people with obesity, to increase clinical utility.

Thus, the findings of the present study suggest that an important focus for obesity treatment is (i) to reduce high‐caloric food cravings that do not occur at eating moments but at random moments throughout the day and (ii) to reduce the variety of high‐caloric food cravings. By learning to control and inhibit urges to eat high‐caloric foods, further weight gain may be prevented and weight may be lost 43. The results of the present study suggest that tailoring treatment to individual high‐caloric food cravings via EMA is feasible. By doing so, ecological momentary interventions could provide therapy directly after a high‐caloric food craving is reported.

## Conflict of interests, source of funding and authorship

The authors declare that they have no conflicts of interest.

This research was funded by grant 12028 awarded to Anne Roefs, from Technology Foundation STW, National Initiative Brain and Cognition [part of the Netherlands Organization for Scientific Research (NWO)], and Philips, under the Partnership program Healthy Lifestyle Solutions.

AR, CN and AJ acquired funding for the study. AR, BB, CN and AJ designed the study. BB collected the data. BB, GS and AR analysed the data. All authors contributed to data interpretation. AR and BB drafted the manuscript. All authors provided feedback and approved the final version of the manuscript submitted for publication.

## Transparency declaration

The lead author affirms that this manuscript is an honest, accurate and transparent account of the study being reported. The reporting of this work is compliant with STROBE guidelines. The lead author affirms that no important aspects of the study have been omitted and that any discrepancies from the study as planned have been explained.
